# Application of the voluntary human approach test on commercial pig fattening farms: a meaningful tool?

**DOI:** 10.1186/s40813-020-00158-y

**Published:** 2020-08-12

**Authors:** Birte Wegner, Ines Spiekermeier, Hendrik Nienhoff, Julia Große-Kleimann, Karl Rohn, Henning Meyer, Heiko Plate, Hubert Gerhardy, Lothar Kreienbrock, Elisabeth Grosse Beilage, Nicole Kemper

**Affiliations:** 1 Institute for Animal Hygiene, Animal Welfare and Farm Animal Behavior, University of Veterinary Medicine Hannover, Foundation, D-30173 Hanover, Germany; 2 Swine Health Service, Chamber of Agriculture in Lower Saxony, D-26121 Oldenburg, Germany; 3 Department of Biometry, Epidemiology and Information Processing, University of Veterinary Medicine Hannover, Foundation, D-30559 Hanover, Germany; 4 VzF GmbH, D-29525 Uelzen, Germany; 5 Marketing Service Gerhardy, D-30826 Garbsen, Germany; 6 Field Station for Epidemiology, University of Veterinary Medicine Hannover, Foundation, D-49456 Bakum, Germany

**Keywords:** Animal welfare, Behavior, Health, Management, Swine

## Abstract

**Background:**

A Voluntary Human Approach Test (VHAT) was performed in pig pens, and relationships between environmental conditions and welfare indicators were investigated. Five variables were measured in 1668 pens in 214 fattening pig herds in Germany: time until the first contact (touching) between a pig and the person in the pen (TUFC), time until the observer was surrounded by pigs within a radius of approximately two meters, percentage of pigs relative to group size [%] surrounding the observer after 1 min (PPSO), percentage of pigs relative to group size [%] that completely avoided contact with the observer during the entire test period, and how the pigs contacted the observer (Score 0 [no touching] - 3 [biting]). Furthermore, variables indicative of the pigs’ environment (e.g., feeding system, ventilation system), management (e.g., number of usable drinkers, number of usable manipulatable materials), and welfare (e.g., tail lesions, ear lesions) were documented.

**Results:**

Pigs engaging in more forceful means of contact (nibbling, biting) approached the observer faster than those exhibiting more gentle types of contact (touching). A lower TUFC was associated with more manipulatable materials present, a higher number of drinkers, and with the control position of the caretaker located inside the pen. Pigs kept in larger groups showed a lower TUFC than those in smaller groups (*P* = 0.0191). However, PPSO was lower in pigs kept in smaller groups (1–12 pigs per pen) with more manipulatable materials available. In groups with low PPSOs, more tail lesions were observed (*P* = 0.0296). No relationship between contact type and tail or ear injuries was detected. In younger pigs, PPSO was higher (49.9 ± 23.2%) than for animals in the second half of the fattening period (45.1 ± 19.9%).

**Conclusions:**

In this on-farm study, the relationships between VHAT behavior and environmental factors revealed that external factors (e.g., management practices, housing conditions) impact animals’ responses to this behavioral test. Therefore, using the VHAT as an animal welfare indicator is valid only if these variables are studied as well.

## Background

In farm animal production, consumers expect food production processes to take animal welfare into account [1–5]. Moreover, because animal welfare is closely connected to health status, stress level, and product quality [6], practices that maximize animal welfare should be carried out on every farm. Ethical issues concerning farm animals’ quality of life should also be taken into account when considering animal welfare [7]. Therefore, strategies for achieving reliable on-farm monitoring of animal welfare are essential [8].

Definitions of animal welfare have been developed by a variety of authors [9–11]. Since animal welfare is a multi-factorial concept, several attempts have been made to define variables that allow a reliable assessment of animal welfare in a given herd [12–14]. However, the reliability of the data generated by measuring these variables is not well established [15, 16]. Animal welfare is multifactorial and complex [17–19]; therefore, the MulTiViS project (“Multivariate Assessment of Animal Welfare through Integrative Data Collection and Validation of Animal Welfare Indicators in Fattening Pigs”) was created to identify key animal welfare variables from a large data pool and to use these to develop a validated animal welfare scoring system for fattening pig herds. As such, the aim of the present study was to determine which variables can be implemented in daily practice.

The animal welfare indicators used in on-farm monitoring should be easily recorded under practical conditions. Including a behavioral test in standardized on-farm animal welfare monitoring procedures necessitates that it is highly repeatable, accurate, and practical. However, the test-retest reliability of behavioral tests is not always given [15, 20], probably due to insufficient standardization or validation [21–23]. Furthermore, there is often little emphasis placed on test specificity, i.e., whether differences between test results are determined by other factors rather than by differences in behavior [23]. As behavior is not only a variable trait but also situation-specific one [24], it is indispensable to check whether the target-variable is sufficiently robust before it is suitable for multivariate and multifactorial analyses. In addition, knowing the influence of the test environment is important to ensure the generalizability of the results [25].

The present study focused on testing the impact of different management and environmental factors on a behavioral test for fattening pigs, the Voluntary Human Approach Test (VHAT). Human Approach Tests are commonly used to evaluate human-animal relationships [24, 26–31]. Corticosteroid levels are higher in pigs with a stronger fear reaction to humans [32–34]. Therefore, a strong fear of humans is associated with a chronic stress response [35]. Behavioral tests have been developed to measure animals’ response to humans, including the Forced Human Approach Test [36, 37], the Human-Animal Relationship Test [13], and the Voluntary Human Approach Test (VHAT) [23, 38, 39]. The VHAT assesses the latency of animals in approaching a motionless human observer [40] and was chosen for the present study because it is less time consuming than the other tests and easy for veterinarians, consultants, and farmers to carry out. Moreover, the VHAT seems to have low inter-observer-variance as the observer is set motionless against a wall and does not carry out any active contact attempts. It has the additional advantage of not requiring any previous animal training.

Since small environmental differences could greatly impact the results of a behavioral test [25, 41], variables such as age, group size, and feeding system must be considered. In the present study, the VHAT was applied at a group level under practical conditions on a large number of farms. The aim of the study was to analyze VHAT outcomes relative to farm management and husbandry practices as well as to established animal welfare indicators (tail and ear injuries). The study was designed to reveal whether the VHAT is well suited to assess animal welfare under highly variable, commercial farming conditions.

## Results

### Descriptive and initial results of statistical analyses of the VHAT variables

The VHAT resulted in data associated with the four continuous variables TUFC, time until the observer was completely surrounded, PPSO, and percentage of pigs relative to group size that completely avoided contact. The descriptive analysis revealed distinct differences between minimum and maximum values as well as extended standard deviations for all of the continuous variables (Table 1). The type of contact had a minimum of 0 and a maximum of 3 and a median value of 2 was observed.

**Table 1:** Descriptive statistics of continuous VHAT variables

VHAT variables	Arithmetic mean^**a**^	Standard deviation	Variation coefficient	Median	Minimum	Maximum
**TUFC [s]**	9.6	11.4	119.5	5.0	0	59
**TUOS [s]**	21.9	15.0	68.6	18.0	1	60
**PPSO [%]**	47.7	21.9	45.9	50.0	0	100
**PPAC [%]**	11.1	12.4	112.2	8.8	0	100

*VHAT* Voluntary Human Approach Test, *TUFC* time until the first contact (touching the observer) of a pig with the person in the pen, *TUOS* time until the observer is completely surrounded by pigs within a radius (of approx. 2 m), *PPSO* percentage of pigs relative to group size [%] surrounding the observer after 1 min, *PPAC* percentage of pigs relative to group size [%] completely avoiding contact with the observer during the entire test period

^a^ study population: 1668 pig groups (pen) from 214 herds

In the linear regression analyses of the continuous variables describing the VHAT, a substantial relationship was determined for TUFC and time until the observer was completely surrounded (*P* <  0.0001; *R*^*2*^ = 0.3004), a moderate relationship for TUFC and PPSO (*P* <  0.0001; *R*^*2*^ = 0.0459), but not for PPSO and time until the observer was completely surrounded (*P* = 0.8378; *R*^*2*^ = 0.0000) and not for the percentage of pigs relative to group size that completely avoided contact and time until the observer was completely surrounded (*P* = 0.0066; *R*^*2*^ = 0.0048) (Table 2). When analyzing the relationships between the categorical variable type of contact and the four continuous variables of the VHAT, the ANOVA revealed significant relationships (*P* <  0.0001) for all four variables concerning the type of contact. Based on these results and the literature, TUFC and PPSO were selected for further in-depth statistical analyses.

**Table 2:** Univariate linear regression analyses (*p*-values and R^2^) of continuous VHAT variables

	TUFC^**a**^	TUOS	PPSO	PPAC
**TUFC**	–	*P* = 0.0001 *R*^*2*^ = 0.3004	*P* = 0.0001 *R*^*2*^ = 0.0459	*P* = 0.0066 *R*^*2*^ = 0.0048
**TUOS**	*P* = 0.0001 *R*^*2*^ = 0.3004	–	*P* = 0.8378 *R*^*2*^ = 0.0000	*P* = 0.5913 *R*^*2*^ = 0.0003
**PPSO**	*P* = 0.0001 *R*^*2*^ = 0.0459	*P* = 0.8378 *R*^*2*^ = 0.0000	–	*P* = 0.0001 *R*^*2*^ = 0.0328
**PPAC**	*P* = 0.0066 *R*^*2*^ = 0.0048	*P* = 0.5913 *R*^*2*^ = 0.0003	*P* = 0.0001 *R*^*2*^ = 0.0328	–

*VHAT* Voluntary Human Approach Test, *TUFC* time [s] until the first contact (touching the observer) of a pig with the person in the pen, *TUOS* time [s] until the observer is completely surrounded by pigs within a radius of approx. 2 m, *PPSO* percentage of pigs relative to group size [%] surrounding the observer after 1 min, *PPAC* percentage of pigs relative to group size [%] completely avoiding contact with the observer during the entire test period

^a^ study population: 1668 pig groups (pens) from 214 herds

**Fig. 1:**
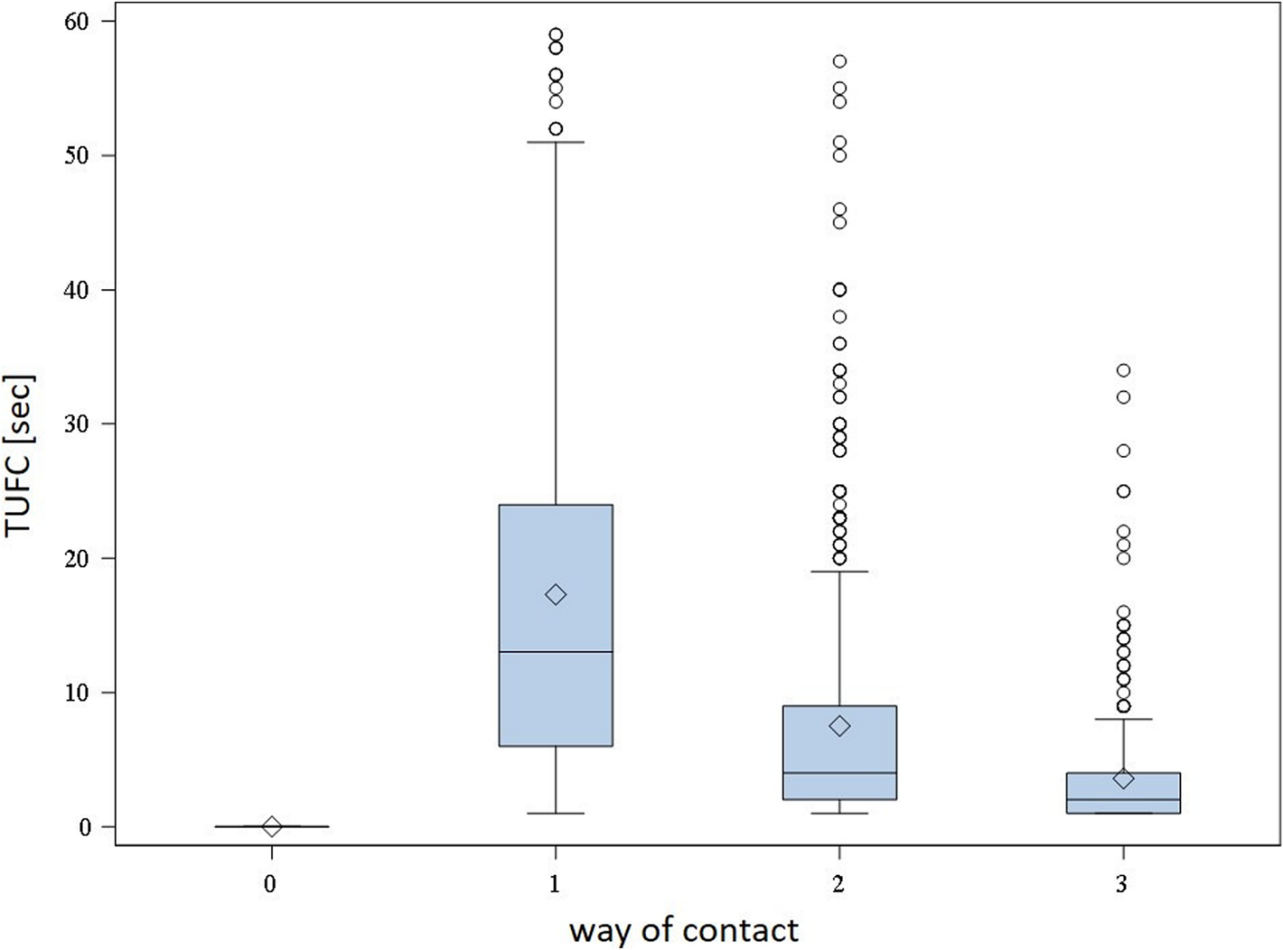
Time [s] until the first direct contact of a pig to the observer (TUFC) categorized by type of contact (0 = no touching, 1 = touching, 2 = nibbling, 3 = biting) of 1657 pens of 214 herds

The relationship between TUFC and the type of contact indicated that groups coming in contact by touching the person needed more time for the first contact (average of 17.3 ± 14.4 s) compared to groups preferring the more intensive types of contact like nibbling (average of 7.5 ± 8.6 s) or biting (average of 3.6 ± 4.5 s) (Fig. 1). The links between PPSO and the type of contact were less clear. At score 0 (no touching) for the type of contact, an average of 12.5 ± 17.2% of the pigs per pen gathered around the observer after 1 min. All scores (1–3) describing direct contact to the observer showed mean values of PPSO differing only by a maximum of 5.1% (score 1: 47.3 ± 20.3%; score 2: 52.3 ± 18.8%; score 3: 52.4 ± 19.4%).

**Fig. 2:**
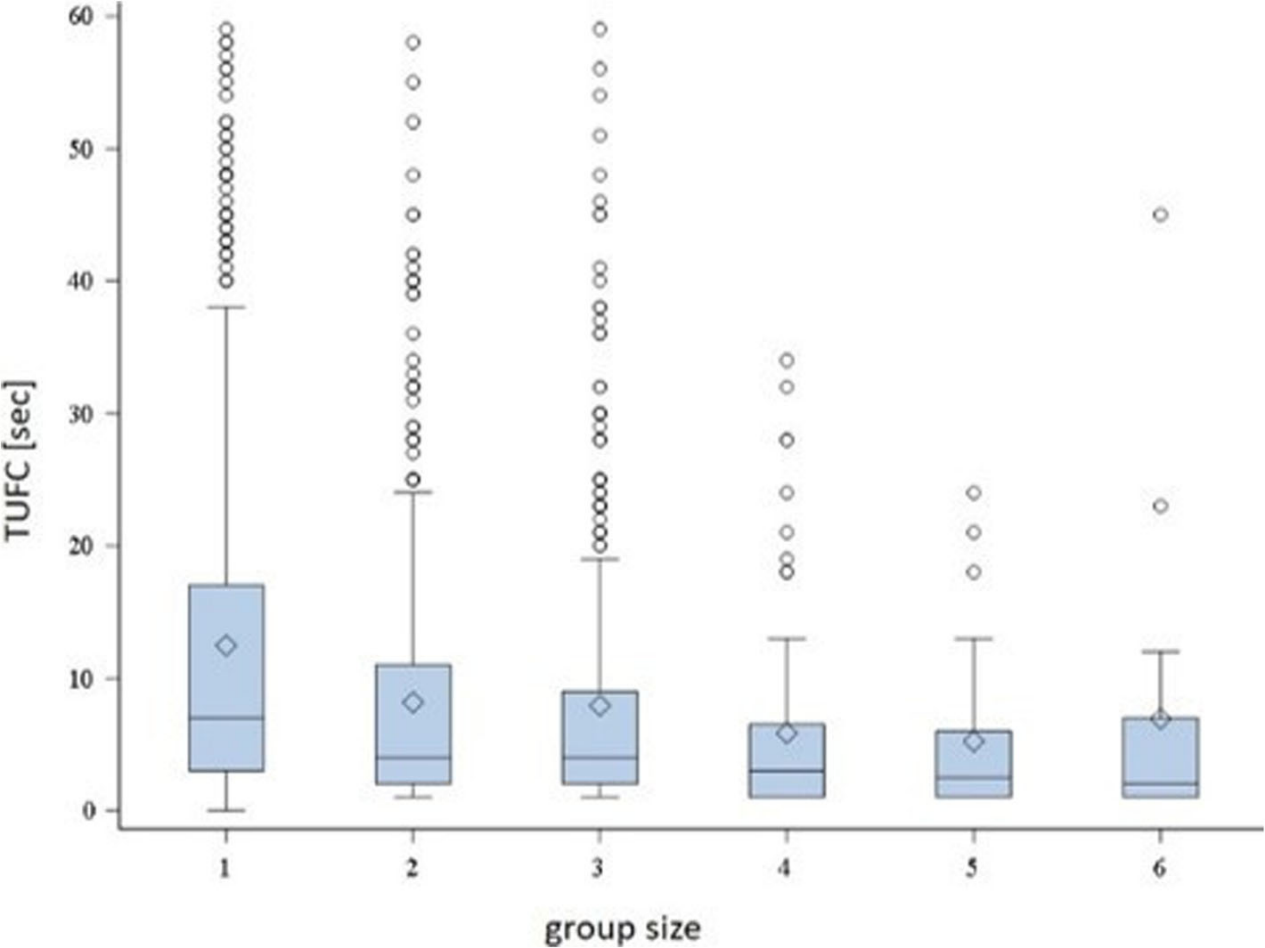
Prevalence of the time until the first contact [s] (TUFC) according to group size (1 = 1–12, 2 = 13–20, 3 = 21–35, 4 = 36–50, 5 = 51–100, 6 > 100 pigs per pen)

In the linear regression analysis, minor relationships were observed between TUFC and the number of animals per pen (*P* <  0.0001, *R*^*2*^ = 0.0106), pigs per usable drinker (*P* = 0.0002, *R*^*2*^ = 0.0091) and loss of ear tissue (*P* = 0.03, *R*^*2*^ = 0.0030). Pigs needed less time (TUFC) to seek contact with the observer when housed in larger groups (Fig. 2), had fewer usable drinkers per pig and fewer pigs with ear tissue loss. From linear regression analysis, nearly all tested variables (number of pigs per pen, pigs per usable manipulatable material, pigs per usable drinker, *Bursa auxiliaris*, skin and tail lesions) showed statistically significant relationships to PPSO (Table 3).

**Table 3:** Univariate linear regression analysis of the time until the first contact [s] (TUFC) and the number of pigs surrounding the observer after 1 min [%] (PPSO) for different animal health-related variables

Variable	TUFC [s]			PPSO [% of pigs per pen]		
	estimator	R-Square	***P***-value	estimator	R-Square	***P***-Value
**number of pigs per pen**	−0.05	0.0106	< 0.0001	− 0.30	0.0842	< 0.0001
**pigs per usable manipulatable material**	0.07	0.0008	0.27	−0.52	0.0125	< 0.0001
**pigs per usable drinker**	−0.18	0.0091	0.0002	−0.39	0.0115	< 0.0001
***Bursa auxiliaris***	−0.09	0.0123	< 0.0001	−0.41	0.0712	< 0.0001
**skin lesions**	−0.11	0.0012	0.1654	−1.04	0.0275	< 0.0001
**ear lesions**	0.07	0.0017	0.1076	−0.14	0.0016	0.0973
**ear substance loss**	0.05	0.0030	0.0302	−0.06	0.0012	0.1590
**tail lesions**	−0.07	0.0025	0.0517	0.14	0.0028	0.0296
**tail substance loss**	−0.06	0.0010	0.2051	0.09	0.0007	0.2810

*TUFC* time [s] until the first contact (touching the observer) of a pig with the person in the pen, *PPSO* percentage of pigs relative to group size [%] surrounding the observer after 1 min

* study population: 1668 pig groups (pen) from 214 herds

**Fig. 3:**
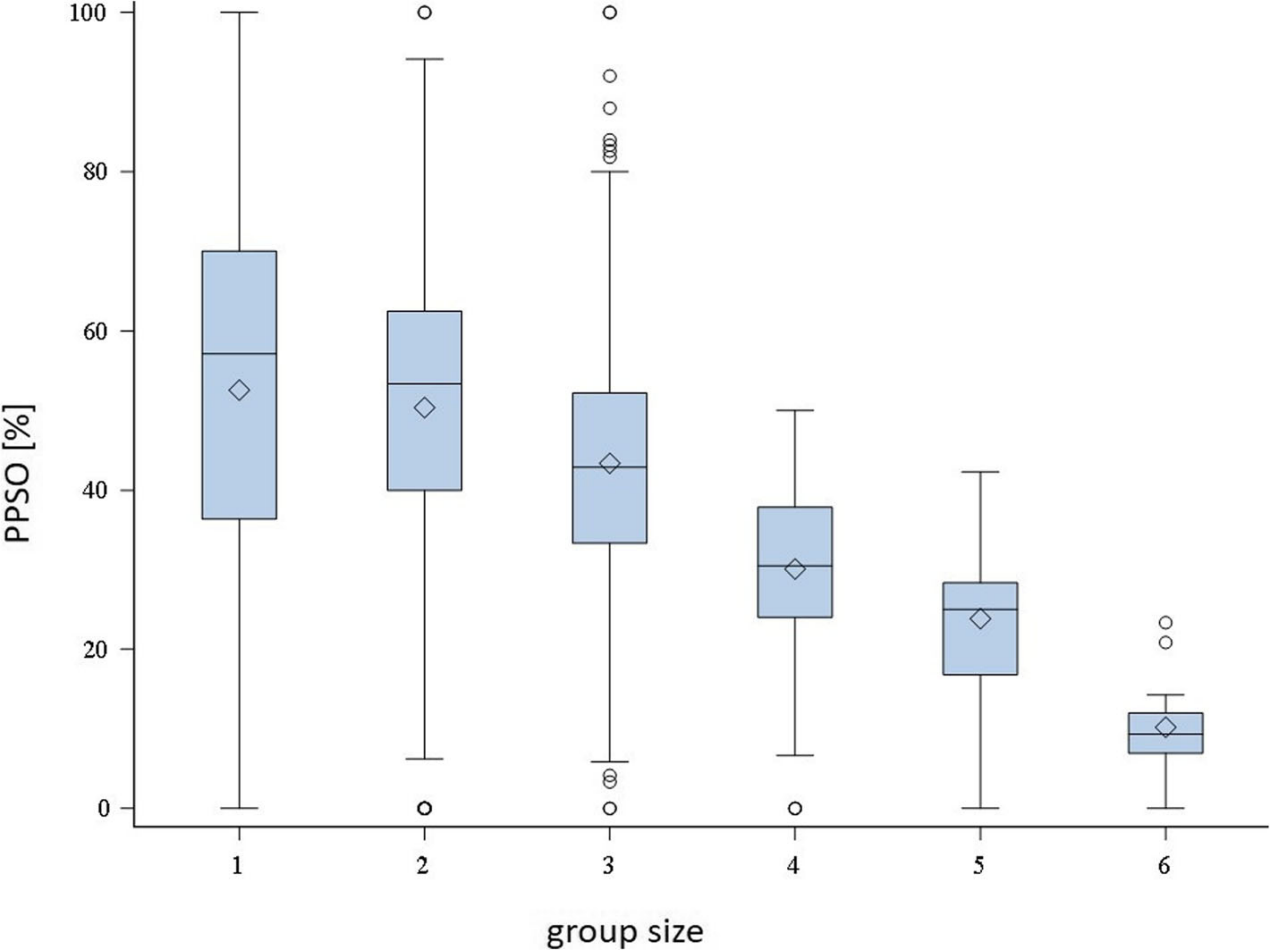
Percentage of pigs relative to group size [%] surrounding the observer after one minute (PPSO) according to group size (1 = 1–12, 2 = 13–20, 3 = 21–35, 4 = 36–50, 5 = 51–100, 6 > 100 pigs per pen)

Pigs surrounded the observer (PPSO) more intensively when housed in smaller groups (Fig. 3) and when more manipulatable materials per pig were available. Groups intensively surrounding the observer had *Bursa auxiliaris* and skin lesions less frequently but tail lesions more frequently.

The analysis of the relationship between the type of contact and the occurrence of bite injuries/ear tissue loss, tail damage or ear necrosis by univariate ANOVA showed a relationship between the type of contact and the occurrence of ear necrosis (*P* = 0.0001). No relationship between the type of contact and bite injuries on the ears (*P* = 0.8313) or tails (*P* = 0.1383) as well as ear tissue loss (*P* = 0.1563) and tail damage (*P* = 0.4440) was found.

### Mixed model

Due to the hierarchical structure of the information, the descriptive results could be biased by the variability between pens on the farms. Therefore, using an overarching model approach, the hierarchies were taken into account by means of mixed linear models (Table 4, Table 5).

**Table 4:** Description and mixed model (MIXED procedure) analysis for the time until the first contact [s] (TUFC) (original and logarithmic data) for different categorical influencing variables

	Description (TUFC)			Model (log (TUFC))				
	n (# pens)	mean [s]	std. dev.	geo. Mean	lower conf. Limit	upper conf. Limit	F-value	***P***-value
**Group size**								
**1 (1–12 pigs per pen)**	578	12.5	13.3	7.1	4.8	14.5	2.72	0.0191
**2 (13–20 pigs per pen)**	452	8.2	9.6	4.7	3.1	8.6		
**3 (21–35 pigs per pen)**	388	7.9	10.1	4.3	2.9	8.5		
**4 (36–50 pigs per pen)**	76	5.9	7.7	3.2	2.1	5.9		
**5 (51–100 pigs per pen)**	30	5.2	6.2	3.0	1.9	5.6		
**6 (> 100 pigs per pen)**	16	6.9	11.7	2.9	2.1	7.1		
**Feeding system**								
**1 (dry feeding)**	25	11.2	10.6	7.0	4.6	12.9	1.27	> 0.05
**2 (wet feeding)**	747	9.1	11.0	5.0	3.3	10.0		
**3 (fully automatic liquid feeding with sensor)**	299	6.9	8.3	4.0	2.6	7.2		
**4 (fully automatic liquid feeding without sensor)**	469	11.9	13.2	6.6	4.5	13.8		
**Ventilation system**								
**1 (aisle ventilation)**	615	10.4	11.5	6.0	4.0	11.7	3.4	0.0025
**2 (perforated air channel)**	494	7.7	9.7	4.1	2.7	8.2		
**3 (diffuse fresh air system)**	43	11.0	11.8	6.7	4.3	12.3		
**4 (high-velocity ventilation)**	174	10.1	12.4	5.6	3.7	10.9		
**5 (slot ventilation)**	161	11.0	13.9	5.3	3.7	12.8		
**6 (others)**	20	12.3	13.6	8.0	4.8	11.8		
**7 (outside pens)**	33	8.1	9.3	4.8	3.0	8.1		
**Age group**								
**1 (1st-6th fattening week)**	842	9.4	11.0	5.2	3.5	10.3	0.3	> 0.05
**2 (7th–12th fattening week)**	698	9.8	11.8	5.2	3.5	10.9		
**Control position of the caretaker pre-fattening period**								
**1 (service corridor)**	1380	10.0	11.6	5.5	3.7	11.2	2.0	> 0.05
**2 (inside the pen)**	131	5.6	8.2	3.1	2.0	5.2		
**3 (others/mixed)**	29	5.3	9.3	2.8	1.8	4.9		
**Control position of the caretaker middle/end- fattening period**								
**1 (service corridor)**	1453	9.9	11.6	5.4	3.7	11.1	1.0	> 0.05
**2 (inside the pen)**	58	3.3	3.4	2.5	1.3	2.6		
**3 (others/mixed)**	29	5.3	9.3	2.8	1.8	4.9		
**Flooring type**								
**1 (straw)**	9	7.3	9.0	3.8	2.6	8.5	0.1	> 0.05
**2 (fully slatted concrete flooring)**	1325	9.6	11.5	5.2	3.5	10.7		
**3 (partially slatted concrete flooring)**	206	9.4	11.2	5.1	3.4	10.2		
**Time of day**								
**1 (08:00–10:59)**	576	9.7	11.1	5.5	3.6	10.8	0.5	> 0.05
**2 (11:00–13:59)**	479	8.9	11.0	5.0	3.3	9.6		
**3 (14:00–16:59)**	384	10.3	12.4	5.3	3.7	11.9		
**4 (17:00–19:59)**	48	9.1	13.0	4.3	3.0	9.8		

* study population: 1668 pig groups (pen) from 214 herds

*TUFC* time [s] until the first contact (touching the observer) of a pig with the person in the pen

**Table 5:** Description and mixed model (MIXED procedure) analysis of the number of pigs surrounding the observer after 1 min [%] (PPSO) according to different categorical influencing variables

	Description			Model			
	n (# pens)	mean [%]	std. dev.	estimator [%]	std. err.	global F-value	global ***P***-value
**Group size**							
**1 (1–12 pigs per pen)**	668	52.6	25.1	43.0	6.0	20.1	< 0.0001
**2 (13–20 pigs per pen)**	472	50.4	19.2	38.9	6.2		
**3 (21–35 pigs per pen)**	400	43.3	15.5	30.8	6.2		
**4 (36–50 pigs per pen)**	80	30.1	11.0	20.7	6.3		
**5 (51–100 pigs per pen)**	31	23.8	9.8	17.8	7.0		
**6 (> 100 pigs per pen)**	17	10.2	5.6	0.5	7.2		
**Feeding system**							
**1 (dry feeding)**	31	42.2	25.1	21.4	9.3	2.2	> 0.05
**2 (wet feeding)**	800	47.5	21.3	29.4	5.1		
**3 (fully automatic liquid feeding with sensor)**	315	42.4	19.1	23.8	5.5		
**4 (fully automatic liquid feeding without sensor)**	522	51.5	23.3	26.5	5.4		
**Ventilation system**							
**1 (aisle ventilation)**	680	48.8	22.7	28.9	6.7	1.2	> 0.05
**2 (perforated air channel)**	526	47.6	20.8	29.2	6.7		
**3 (diffuse fresh air system)**	45	36.7	20.7	23.6	7.8		
**4 (high-velocity ventilation)**	188	49.8	22.5	27.2	6.7		
**5 (slot ventilation)**	175	47.5	21.3	24.7	7.0		
**6 (others)**	21	34.9	14.2	18.8	9.1		
**7 (outside pens)**	33	38.9	18.9	24.5	6.2		
**Age group**							
**1 (1st-6th fattening week)**	908	49.9	23.2	26.7	5.7	5.1	0.0237
**2 (7th–12th fattening week)**	760	45.1	19.9	23.9	5.8		
**Control position of the caretaker pre-fattening period**							
**1 (service corridor)**	1501	48.4	21.8	non-est	.	1.0	> 0.05
**2 (inside the pen)**	135	42.8	22.2	non-est	.		
**3 (others/mixed)**	32	33.7	19.3	non-est	.		
**Control position of the caretaker middle/end- fattening period**							
**1 (service corridor)**	1574	48.2	21.7	non-est	.	1.1	> 0.05
**2 (inside the pen)**	62	40.5	23.8	non-est	.		
**3 (others/mixed)**	32	33.7	19.3	non-est	.		
**Flooring type**							
**1 (straw)**	9	20.4	7.6	24.6	12.9	0.8	> 0.05
**2 (fully slatted concrete flooring)**	1433	47.8	21.8	26.9	4.3		
**3 (partially slatted concrete flooring)**	226	48.2	22.3	24.4	4.5		
**Time of day**							
**1 (08:00–10:59)**	619	47.4	21.8	27.8	5.7	1.8	> 0.05
**2 (11:00–13:59)**	509	48.5	21.4	27.6	5.6		
**3 (14:00–16:59)**	423	47.3	22.5	27.7	5.9		
**4 (17:00–19:59)**	63	43.2	25.9	18.1	7.0		
**Temperature**							
**1 (< 15 °C)**	10	49.8	17.5	32.5	14.6	0.7	> 0.05
**2 (15–19.9 °C)**	245	46.1	19.8	21.8	5.1		
**3 (20–22.9 °C)**	780	47.7	21.3	23.0	4.8		
**4 (23–26 °C)**	436	49.5	24.2	24.8	4.9		
**5 (> 26 °C)**	181	44.5	21.5	24.3	5.0		

*PPSO* percentage of pigs relative to group size [%] surrounding the observer after one minute)

* study population: 1668 pig groups (pen) from 214 herds

Log (TUFC) was related to group size (*P* = 0.0191; *F* = 2.72) (Table 4), as the time until pigs came in contact with the observer was shorter in larger groups (e.g., 51–100 pigs per pen, average 5.2 ± 6.2 s) than in smaller groups (e.g., 1–12 pigs per pen, average 12.5 ± 13.3 s). For log (TUFC) (*P* = 0.0025; *F =* 3.4), an association with the ventilation system was also found. Pigs in pens with a perforated air channel had the shortest TUFC (average 7.7 ± 9.7 s). Log (TUFC) was also related to the number of pigs per usable manipulatable material (*P* = 0.0099). The shorter the TUFC, the more usable manipulatable materials were available to the pigs. For the type of flooring, time of day, feeding and ventilation system, and position of the caretaker, no statistically significant effects were identified (*P* >  0.05; *F =* 2.0).

PPSO was influenced by group size (*P* <  0.0001; *F =* 20.1) (Table 5). In small groups (e.g., 1–12 pigs per pen, average 52.5 ± 25.1%), more pigs were in the 2-m radius around the observer than in larger groups (e.g., > 100 pigs per pen, average 10.2 ± 5.6%). Statistically significant effects were also found for age group (*P* = 0.0237; *F =* 5.1). PPSO was higher in pigs in age group 1 (average 49.9 ± 23.2%) than in age group 2 animals (average 45.1 ± 19.9%). For the position of the caretaker, feeding and ventilation system, and flooring type, no statistically significant relationships were found (*P* >  0.05; *F =* 1.1).

## Discussion

The present study examined whether the VHAT is an appropriate and practical method for gathering information on pig welfare under diverse on-farm conditions. A large sample of fattening farms was included and extensive data were collected. In general, indicators or tests of animal welfare should provide meaningful and valid information that is not influenced by factors not directly related to animal welfare. Robust relationships with other well-established welfare indicators (e.g., skin and tail lesions) help determine the suitability of newly applied tests. Therefore, in the present study, associations between different environmental factors and animal health characteristics and VHAT results were analyzed from over 200 pig fattening farms to provide novel insight into the applicability of this behavioral test for on-farm animal welfare assessment.

The Human Approach Test has been used to evaluate the behavior of individual pigs for close to 40 years [23, 32, 42–45]. These previous studies focus primarily on identifying personal traits that describe an individual’s ability to deal with different challenges [44]. For this purpose, the animals are usually housed in specially prepared experimental units and separated from their group during the test period. Studies in which the Human Approach Test is performed at the group level are rare [30, 46, 47]. The VHAT has been the focus of a few studies conducted on-farm at the group level [39, 48]. To avoid social isolation, which is a stress factor on the animals during a test [23], the pigs in the present study were tested in their familiar environment and group.

The sample population was representative of fattening pig farms in North-West Germany and a real, practical on-farm, rather than experimental, environment was used. To avoid observer bias, all farms were visited by the same team of two persons.

The results of the present study can in part be compared with those from other studies that apply the VHAT [30, 39, 48]. Nonetheless, there are no comparative values for all five variables used here to characterize this test. The pigs in the study groups generally approached the observer fast (TUFC) (9.6 ± 11.4 s) and in a similar period as previously described with mean latency times in fattening pigs at the group level between 0 and 10.4 s [30, 39, 48]. The pigs surrounded the observer after 21.9 ± 15.0 s and about 11.1 ± 12.4% of the group avoided contact with the observer. No comparative values have been published for those two VHAT variables to date. A total of 47.7 ± 21.9% of the group gathered around the observer (PPSO) within a radius of 2 m, which is comparable to the 33% surrounding the observer within a radius of a pig’s length reported by Veit et al. [39].

In addition to the individual variables, relationships between variables were also considered, such as the relationship between TUFC and type of contact. The time pigs needed to come into contact with the observer positively associated with the intensity of the contact. However, comparable data on the type of contact have not been published yet. It is likely that pigs, which are generally very curious animals, that approach the observer quickly also display more pronounced exploratory behavior, using a more intensive type of contact to explore an object/human. This assumption explains the connection between TUFC and type of contact. Since comparative values from other studies are available for TUFC and PPSO [30, 39], these two parameters were selected for further statistical analyses.

To avoid any potential confounding bias on the VHAT-associated measures, the confounding effects of a series of additional variables were considered. Concerning the PPSO, significant differences were found between the two age groups. In age group 1, the PPSO was higher than in age group 2. However, the difference in the descriptive mean PPSO according to age groups was only 4.8% (AG 1 49.9 ± 23.2%; AG 2 45.1 ± 19.9%). This result contradicts the aforementioned assumption that pen size affects the variables describing the VHAT, as space was more limited for pigs in age group 2 than for the younger animals. Other studies suggest another explanation for the effect of age by demonstrating that animals become more experienced [49] and less fearful [23] with increasing age. This effect could also be due to changes in the stable or other management factors that were not included in the present analysis. Therefore, some residual confounding may have occurred. In general, the interpretation of VHAT results should always include variables that influence fear of humans. Even though the human-animal relationship is reduced in commercial husbandry in comparison to traditional husbandry systems, it is still important [45] and may influence the behavior measured by the VHAT. Breeding sows for example supervised by farmers trained in their manner and behavior tended to retreat less before an observer approached [50]. In contrast, female pigs that had unpleasant experiences with humans (e.g. short stamping) were more afraid of approaching an observer than pigs that had pleasant experiences (e.g. stroking) [51].

Given that ear and tail biting behavior, in addition to stress, frustration, and dominance structures, is associated with aggression within pig groups [52–54], the type of contact was assumed to be more intensive in groups showing typical lesions. Also, the hypothesis that pigs who approach humans faster have more body lesions [30] was not confirmed by this study. In another study, no significant association between tail biting and behavior measured by VHAT at the group level was found [39]. In the present study, the PPSO rose with increasing number of tail lesions. This finding supports observations that groups in which tail biting occurs generally show more redirected explorative behavior [55]. However, PPSO was the only variable of the VHAT to show this result. Tail biting itself is an indicator of reduced animal welfare [56]. If the VHAT is indicative of animal welfare, a link or association of the VHAT results to other established animal welfare indicators (e.g., tail lesions) would be expected. The relationship between the PPSO and tail lesions seems important here: there is a relationship between the VHAT and a direct and easy-to-measure health indicator. If, in this case, the VHAT and/or the established indicator are inaccurately measured, this would be deemed a non-differential information bias, which, in turn, would disguise the effect so that it would go undetected under practical conditions. Since the target variables are considerably influenced by the accompanying variables, recording variables alone only provides limited information about animal welfare. Therefore, it is important to include the corrected recording along with the estimators of the influence variables from an overarching model in an evaluation to consider these confounders.

Data description as a whole can be prone to confounding bias, if other direct and indirect animal welfare indicators are linked to the investigated VHAT variables. In addition, if pen results on any given farm vary, a supplementary bias may be introduced. Therefore, a final evaluation of the VHAT variables was performed that takes this into account using a mixed model with a fixed multifactorial assessment of confounders and random hierarchical factors from pens on any one farm. From a methodical point of view, this strategy is crucial [57]. On the other hand, direct on-site interpretation is hindered. This may underlie the absence of comparable published results.

The results of the statistical analyses revealed clear relationships between the outcome of the VHAT and factors assigned to the farm environment and management. TUFC and PPSO were statistically relative to group size, usable manipulatable material, number of usable drinkers, feeding system, and position of the caretaker when inspecting the animals.

Concerning environmental factors, the number of pigs per pen significantly correlated with the TUFC and PPSO. Pigs approached the human observer faster in larger groups. These results are supported by other studies: pens with seven to eight pigs had longer latency periods (10.4 s) [30] than pens with 35 to 40 pigs (0 s) [39]. Group dynamics may induce behavioral patterns (negatively or positively) that an individual animal separated from its group would not so quickly show. The effect of group size on PPSO was contrary to TUFC. The smaller the group, the more animals were within the 2-m radius around the observer. This effect can be explained by the part of the pen included in the 2-m radius around the observer. In smaller pens, the radius around the observer covered a larger area of the pen compared to larger pens. Therefore, pigs might more readily choose to enter the 2-m radius in smaller pens.

Relationships between the TUFC and PPSO were identified for several environmental factors including the feeding system, ventilation system, number of usable drinkers, number of usable manipulatable materials, and position of the caretaker. To our knowledge, this is the first time that data describing on-farm variables have been put in the context of the outcome of a behavioral test. On the one hand, the test for single effects on the outcome of behavioral tests requires other experimental study designs, but this was not the aim of the present study. On the other hand, environmental factors cannot be ignored when discussing the application of tools for on-farm animal welfare assessment. Statistically significant relationships were detected between TUFC and the ventilation system. As data on the impact of different ventilation systems on certain behaviors are lacking, we can only speculate in which way this relationship is conceivable. For example, pigs’ behavior can be directed with regard to the acceptance and setting up of functional areas by targeted airflow in pens and by avoiding drafts [58]. Regarding the amount of usable manipulatable materials, statistically significant relationships with TUFC were also found. Even though published data on such relationships are not available, a logical conclusion can be drawn in this context. In the present study, the pigs came into contact with the observer more quickly when more usable manipulatable materials were available in the pen. This could be explained by the fact that animals that compete less for manipulatable materials are more relaxed and the more relaxed the overall situation in the group is, the more time the individual animal might have to exert individual behaviors.

## Conclusion

In summary, this study showed that VHAT may be applied on-farm to pig populations under practical, diverse conditions. Since VHAT values as target measures for animal welfare are significantly influenced by accompanying variables (management, health, etc.), these variables should not only be recorded, but included in welfare evaluations. It is essential to include the relationships or influences of the variables to one another in the evaluations as well as the population variation. In addition, the variation in the VHAT on any one farm, e.g., in different pens, must be taken into account when an on-farm evaluation is carried out.

A direct connection between VHAT and animal welfare indicators could only be established for tail lesions. This suggests that it is possible to evaluate animal welfare on the basis of variables collected under practical conditions. To establish VHAT as an independent animal welfare indicator, more precise protocols would enable valid assessment of animal welfare under practical conditions that takes into account confounders and reduces information bias by means of more accurate definitions.

## Methods

### Herd selection and characteristics

The study was performed between November 2017 and September 2018 on 214 commercial fattening pig herds in Lower Saxony, Germany. All farmers were members of the VzF GmbH (Uelzen, Germany) and participated in the study on a voluntary basis. The study population consisted of 23 farrow-to-finish and 191 fattening herds. The pig places ranged from 160 to 3250 (lower quartile: 650, median: 1050, upper quartile: 1440) fattening pigs.

### Animal health, husbandry, and management variables

Each farm was visited once by two trained veterinarians. First, at the beginning of each farm visit, an interview was conducted with the farmer regarding the general management of the herd. Farmers were asked about management variables such as the number of daily inspections of the pigs and the control position of the caretaker during the pre- and middle/end- fattening period (from the service corridor or by entering the pen). Subsequently, depending on the size and structure of the farm, one to eight pens per farm were randomly selected and evaluated following a standardized protocol (modified according to [59]). Pen selection was determined by the structure of the farms. For example, if a farm had only four pens, all four pens were selected for the assessment using the Kish method [59]; however, if the farm had 20 pens, eight pens were randomly selected. The study included 214 pig herds, a total of 1668 pens, and 33,668 pigs to record factors related to animal health, husbandry, and management. Mixed groups of females and surgically castrated males were kept in 89.2% of the pens, only boars in 8%, and, in 2.8% boars, castrated males, or females were housed together. The pigs were usually moved at approximately 10 weeks old to the fattening units, with the exception of 10 farms that started fattening pigs at a younger age. On average, the animals were slaughtered around 25 weeks of age. The pigs from different farms varied according to group size (15 median pigs per pen, min 5, 25% quantile 11, 75% quantile 23, max 290), age (age group 1 = pigs in the first half of fattening, age group 2 = pigs in the second half of fattening), and genetics (e.g. DanBred, PIC, BHZP and mixed genetics). With the exception of 39 farms where boar fattening was practiced exclusively, male pigs were surgically castrated. Almost all pigs had docked tails, in accordance with the tail docking derogation granted to these farms.

Most of the pens (85.9%) were equipped with fully slatted concrete flooring, 13.6% with partially slatted concrete flooring, and 0.5% with straw bedding. The average stocking density was 0.82 ± 0.12 m^2^ per pig. The stables were equipped with various feeding and ventilation systems. The average compartment temperature during the study period was 22.2 ± 2.5 °C with an average relative air humidity of 69.8 ± 14.3%.

Animal health-related variables included bite injuries and loss of ear tissue and tail damage (percentage of pigs per pen), ear necrosis (percentage of pigs per pen), *Bursa auxiliaris* (percentage of pigs per pen), and superficial skin injuries (percentage of pigs per pen with > 10 superficial lesions). Evaluations were carried out using a yes/no scale. Thus, the presence of such findings was recorded for each animal per pen, not the amount per animal.

Variables characterizing the pigs’ environment were group size, feeding system, ventilation system, number of pigs per usable manipulatable material, and number of pigs per usable drinker. Manipulatable materials were deemed usable when supplied at a height where the animals could access them and they were in working condition (e.g., a filled straw rack). A drinker was only considered usable if it was fully functioning and positioned at a height that the animals could reach.

### Voluntary Human Approach Test (VHAT)

Depending on the structure and size of the fattening pig herd, the VHAT was carried out in up to eight randomly chosen pens per farm (Kish grid method modified from [59]). The VHAT was always performed by the same two observers in the pigs’ home pens (*n* = 1668). A modified form of the VHAT was applied in accordance with [40, 47].

The test began when the first observer entered and then walked through the pen once to force all of the pigs to stand up. Next, the observer leaned motionless against the wall of the pen opposite the service corridor, while a second observer recorded the pigs’ reactions from the corridor. No modifications were made to the conditions or materials in the pen, so to conduct the test in an environment familiar to the animals. Both observers were unknown to the animals.

Five variables were assessed during the one-minute observation period:

1. The time until first contact (TUFC) (touching the observer) of a pig with the person in the pen.
2. The time until the observer was surrounded by pigs within a radius of approximately two meters.
3. The percentage of pigs relative to group size [%] surrounding the observer (PPSO) after 1 min.
4. The percentage of pigs relative to group size [%] that completely avoided contact with the observer during the entire test period.

## The type of contact the pigs had with the observer based on a four-scale score (0 = no touching, 1 = touching, 2 = nibbling, 3 = biting).

### Statistical analysis

All results were stored for the entire MulTiViS study in a relational SQL-database exclusively computed for this study that took into account all hierarchical levels on the farms. With a PHP web-interface set up on the data-protected virtual server of the University of Veterinary Medicine, Hannover, data entry and plausibility were organized by formal format checks and basic data description for investigated variables. Statistical evaluations were carried out using SAS-Software (9.4 m5 as well as the Enterprise Guide Client 7.15, SAS Institute Inc., Cary, NC, USA). The unit of the statistical analysis was the pen and all VHAT results were based on groups of pigs housed in their respective pens.

Data were checked for normality and, for further analysis, the right-skewed data of TUFC were transformed into their logarithmical form [log (TUFC)] prior to calculations to meet the criteria of normal distribution. The Kolmogorov-Smirnov test was used to assess model residuals for normal and lognormal distribution. Q-Q plots were assessed visually.

Based on the assumption that the variation between farms exceeds the variation within pens, a non-standardized description of pen data was carried out first. To describe the variables under investigation, linear regression analyses (REG procedure) were performed to evaluate the relationship between the different continuous variables of the VHAT (all except for the categorical variable type of contact). The relationship between the type of contact and the continuous variables was determined using an analysis of variance (ANOVA). Based on this analysis and a review of the literature, TUFC and PPSO were selected and their relationship to the number of animals per pen, pigs per usable manipulatable material, pigs per usable drinker, pigs with *Bursa auxiliaris*, superficial skin lesions, and lesions and loss of ear tissue and tail damage was described using linear regression analyses (REG procedure).

Statistical inference was used to ensure all of the components of variation were taken into account using multi-factorial mixed model analyses of variance with fixed and random effects (MIXED procedure with the Kenward-Roger degree of freedom method). First, the model was calculated with all potential influencing variables. Second, a backward selection was performed including variables with *P* <  0.5 in the model only. The model considered age group, group size, feeding system, ventilation system, flooring type, pigs per usable manipulatable material, pigs per usable drinker, control position during the pre- and middle/end-fattening period, number of daily inspections, and time of day as fixed effects to detect statistically significant differences in both log (TUFC) and PPSO associated with the environmental factors under consideration. Pens within the farms as subjects were considered to be random effects. The general model’s level of significance was *P* <  0.05 with no adjustment for multiple testing.

## Data Availability

Our data are available upon request, but only in exceptional cases. The data were collected on an individual basis from farmers. Each participant gave written consent with the understanding that data would not be transferred to any third party. Therefore, any data transfer to interested persons is not allowed without an additional formal contract. Data are available to qualified researchers who sign a contract with the University of Veterinary Medicine Hannover. This contract will include guarantees to maintain data confidentiality in accordance with the provisions of the German Data Protection Law. Currently, there exists no data access committee or other body that could be contacted for the data because there has been no need until now. Interested cooperative partners who are considering sharing a contract as described above may contact: Prof. Dr. Lothar Kreienbrock, Department of Biometry, Epidemiology and Information Processing University of Veterinary Medicine Hannover, Buenteweg 2, D-30559 Hannover, Germany; e-mail: lothar.kreienbrock@tiho-hannover.de.
